# Supplementary material for: Enantioselective Synthesis and Pharmacological Evaluation of Aza-CGP37157–Lipoic Acid Hybrids for the Treatment of Alzheimer’s Disease
Source: Antioxidants (Basel). 2022 Jan 4;11(1):112. doi: 10.3390/antiox11010112 (PMC8772772; doi:10.3390/antiox11010112)
Supplement: Supplementary file 1 [file antioxidants-11-00112-s001.zip › antioxidants-1505194-supplementary.pdf]

antioxidants

Supplementary Material: Enantioselective Synthesis and Pharmacological Evaluation of Aza-CGP37157–Lipoic Acid Hybrids for the Treatment of Alzheimer's Disease

Ángel Cores [1,†], Patrycja Michalska [2,†], José Miguel Pérez [1], Enrique Crisman [3,4], Clara Gómez [4], Mercedes Villacampa [1], José Carlos Menéndez [1,*] and Rafael León [4,*]

[1] Unidad de Química Orgánica y Farmacéutica, Departamento de Química en Ciencias Farmacéuticas, Facultad de Farmacia, Universidad Complutense, 28040 Madrid, Spain; acores@ucm.es (Á.C.); josemigp@ucm.es (J.M.P.); mvsanz@ucm.es (M.V.)
[2] Department of Chemistry, Imperial College London, SW7 2BX London, UK; p.dziama@imperial.ac.uk
[3] Instituto de Investigación Sanitaria, Hospital Universitario de la Princesa, 28006 Madrid, Spain; ecrisman@outlook.com
[4] Instituto de Química Médica, Consejo Superior de Investigaciones Científicas (IQM-CSIC), 28006 Madrid, Spain; clara.gomezserrano@estudiante.uam.es
* Correspondence: josecm@ucm.es (J.C.M.); rafael.leon@iqm.csic.es (R.L.)
† These authors contributed equally.

Table S1. Nrf2 induction of compounds in the AREc32 cell line expressed as CD values. AREc32 cells were treated with increasing concentrations (0.3, 3, 10 and 30 μM) of the corresponding compound for 24 h and thereafter, luciferase activity was measured. Data are expressed as the concentration required to double the specific luciferase activity (CD).

Entry	Compound	CD (μM)
1	**CGP37157**	-[2]
2	***S*-Lipoic acid**	>600
3	***R*-Lipoic acid**	>600
4	***rac*-3a**	14.8 ± 1.6[3]
5	**(*rac*,*R*)-3b**	9.8 ± 1.6[3]
6	**(±)-6**	> 30[2]
7	***R*-6**	11.8 ± 0.5
8	***S*-6**	14.4 ± 0.2
9	**(*S*,*S*)-7a**	26.5 ± 1.3
10	**(*S*,*R*)-7b**	11.9 ± 1.3
11	**(*R*,*S*)-7c**	> 30
12	**(*R*,*R*)-7d**	> 30

Data are means ± SEM of at least five different experiments in duplicate.

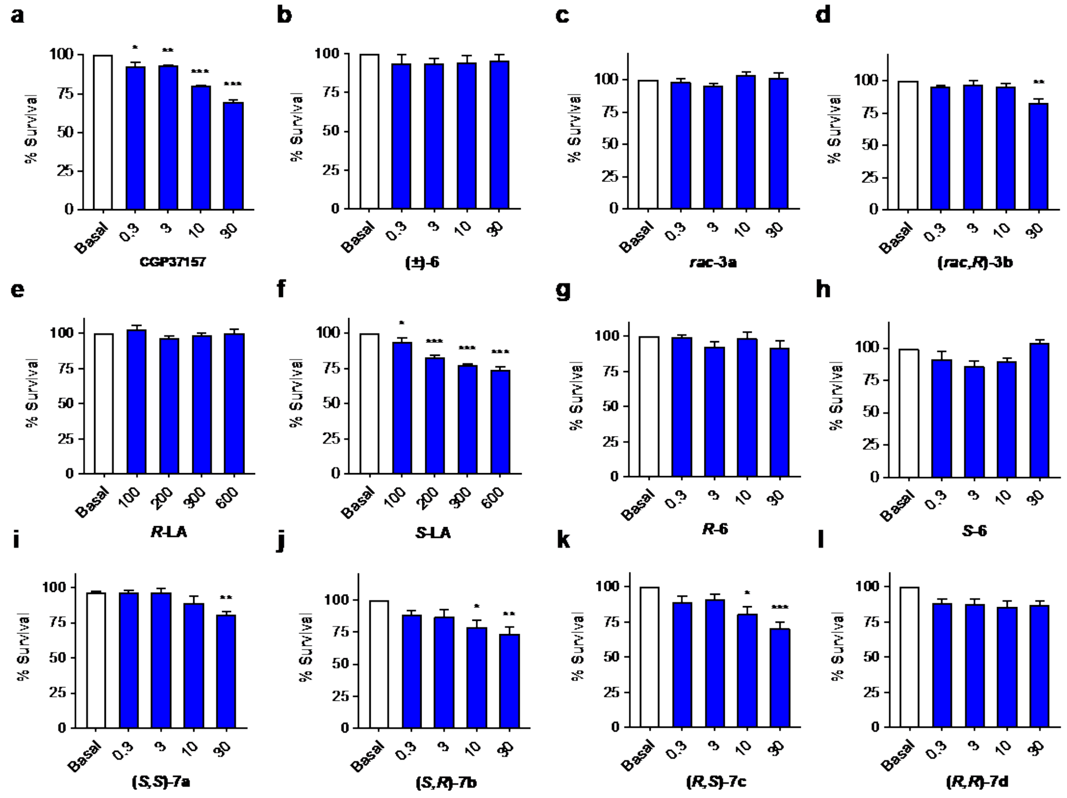

Figure S1. Cytotoxicity of CGP37157, **3**, **6**, LA and **7** in the AREc32 cell line. AREc32 cells were treated with each compound at desired concentration (0.3, 3 10 and 30 μM) for 24 h. Thereafter, cell viability was assessed by the MTT method. Data are means of five different cultures in duplicate. **p* < 0.05, ***p* < 0.01, ****p* < 0.001 compared to basal.

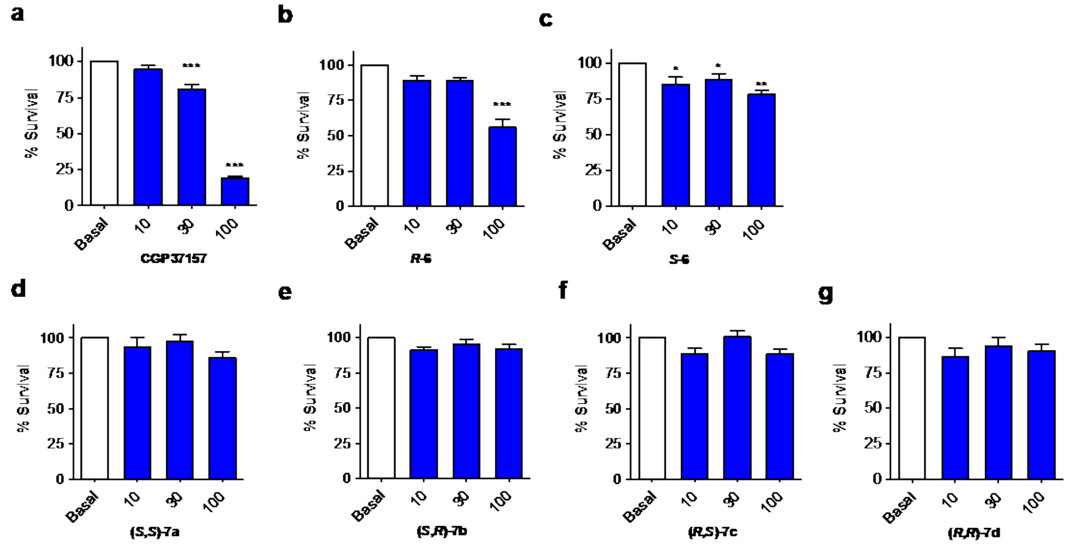

Figure S2. Cytotoxicity of CGP37157, **6** and **7** in the SH-SY5Y cell line. SH-SY5Y cells were treated with increasing concentrations of each compound (10, 30 and 100 μM) for 24 h. Thereafter, cell viability was assessed by the MTT method. Data are means of three different cultures in duplicate. **p* < 0.05, ***p* < 0.01, ****p* < 0.001 compared to basal.

Figure S3: WB original images

HO-1
β-actin

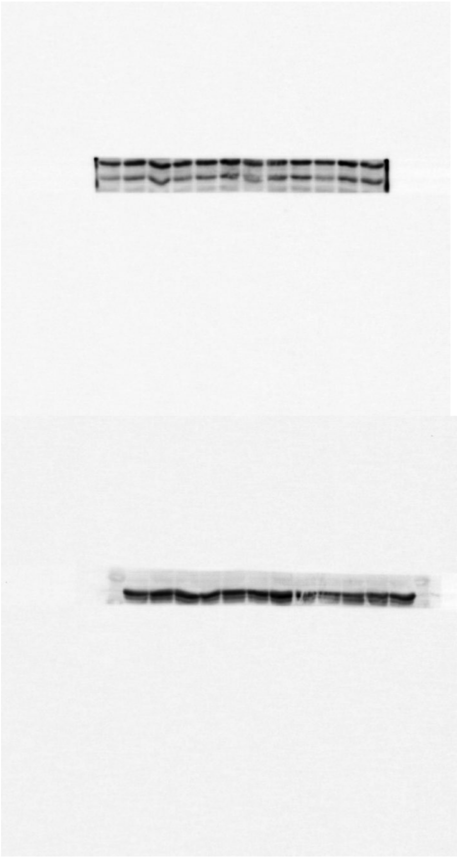

GCLc
β-actin

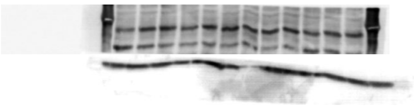

Table S2. Neuroprotective effect of compounds in SH-SY5Y cells against the toxic combination R/O. Compounds were pre-incubated (1 μM) for 24 h, then media was replaced for fresh medium containing compounds and R/O (30/10 μM) for another 24 h. Viability was assessed by the MTT method.

Entry	Compound	% Survival	% Protection
1	**Basal**	100.0	
2	**R/O**	53.4 ± 6.3$^{\#\#\#}$	
3	**Mel**	68.0 ± 1.5	46.1**

7	**CGP37157**	76.2 ± 5.7	46.2***
4	***rac*-3a**	69.0 ± 5.6	52.1**
5	**(*rac,R*)-3b**	72.9 ± 3.5	59.3**
6	**(±)-6**	74.4 ± 4.1	44.1***
8	***R*-6**	75.1 ± 2.9	45.7***
9	***S*-6**	74.7 ± 2.5	51.5***
10	**(*S,S*)-7a**	74.8 ± 3.2	43.4***
11	**(*S,R*)-7b**	81.0 ± 4.7	61.1***
12	**(*R,S*)-7c**	76.1 ± 2.9	47.2***
13	**(*R,R*)-7d**	77.6 ± 4.4	50.7***

Data are means of five different cultures in duplicate. ###p < 0.001 compared to basal; **p < 0.01, ***p < 0.001 compared to R/O.

Table S3. Neuroprotective effect of compounds in SH-SY5Y cells against OA. Compounds were pre-incubated (1 μM) for 24 h, then media was replaced for fresh medium containing compounds and OA (30/10 μM) for another 24 h. Viability was assessed by the MTT method.

Entry	Compound	% Survival	% Protection
1	**Basal**	100.0	
2	**OA**	54.7 ± 4.7###	
3	**Mel**	70.3 ± 4.3	31.1*
5	**CGP37157**	78.8 ± 6.1	56.3**
4	**(±)-6**	69.1 ± 6.4	33.4*
6	***R*-6**	59.3 ± 10.6	14.2
7	***S*-6**	71.5 ± 10.0	41.9*
9	**(*S,S*)-7a**	63.2 ± 7.6	21.2
8	**(*S,R*)-7b**	70.6 ± 10.3	40.2*
11	**(*R,S*)-7c**	50.7 ± 15.6	n.p$
10	**(*R,R*)-7d**	63.3 ± 8.7	21.0

Data are means of five different cultures in duplicate. ###p < 0.001 compared to basal; **p < 0.01, ***p < 0.001 compared to R/O. n.p$ compound does not protect cells.

Table S4. Calculated and experimental elemental analysis.

Compound	Formula	Calculated				Found			
		%C	%H	%N	%S	%C	%H	%N	%S
**1**	$C_{17}H_{18}Cl_2N_2OS$	55.29	4.91	7.59	8.68	55.06	4.97	7.71	8.82
**2**	$C_{17}H_{20}Cl_2N_2OS$	54.99	5.43	7.54	8.63	55.57	5.67	7.30	8.77
**3**	$C_{13}H_{12}Cl_2N_2$	58.45	4.53	10.49	--	58.28	4.39	10.79	--
**4**	$C_{15}H_{16}Cl_2N_2O$	57.89	5.18	9.00	--	55.10	4.91	8.35	--

**5**	$C_{19}H_{17}BrCl_2N_2O_3$	48.33	3.63	5.93	--	48.18	3.52	5.82	--
**6**	$C_{17}H_{16}Cl_2N_2O_2$	58.14	4.59	7.98	--	58.46	4.72	7.74	--
**7**	$C_{25}H_{28}Cl_2N_2O_3S_2$	55.65	5.23	5.19	11.88	55.47	5.08	5.16	11.75

Copies of NMR spectra

(*R*,*Z*)-*N*-((2-Amino-5-chlorophenyl)(2-chlorophenyl)methylene)-2-methylpropane-2-sulfinamide (***R*-1**)

^{1}H NMR (250 MHz, CDCl3)

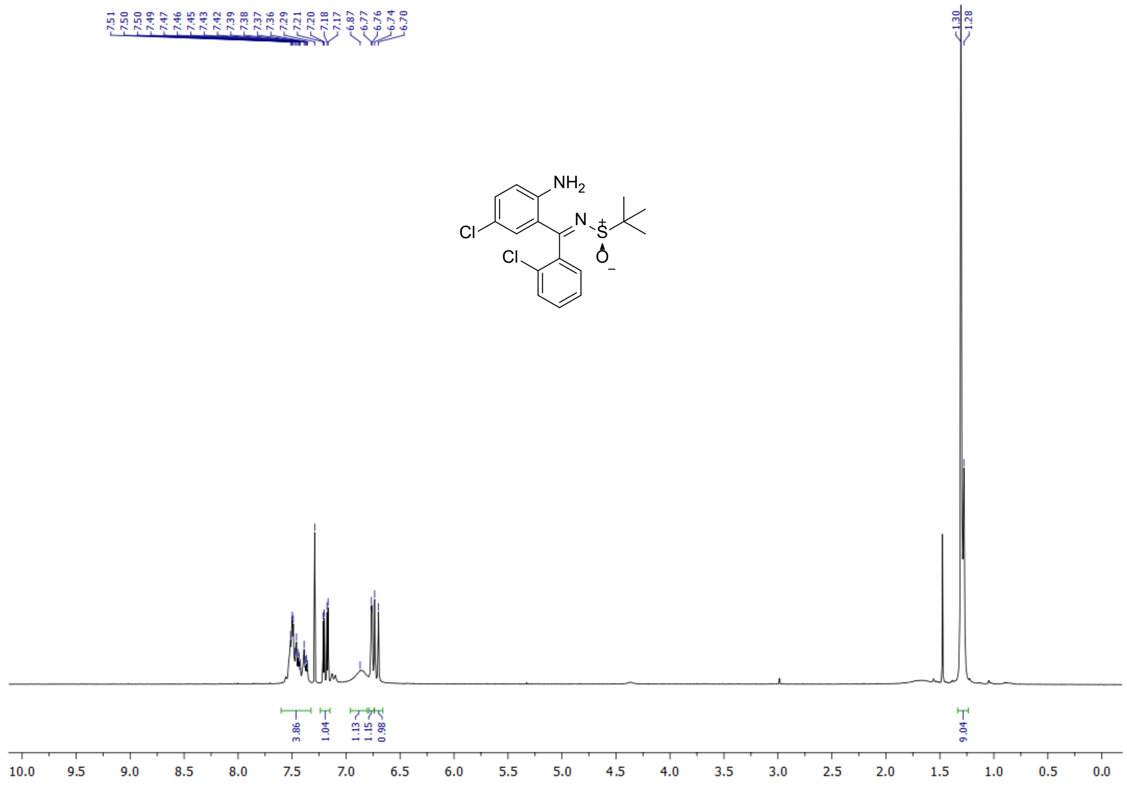

^{13}C NMR (63 MHz, CDCl3)

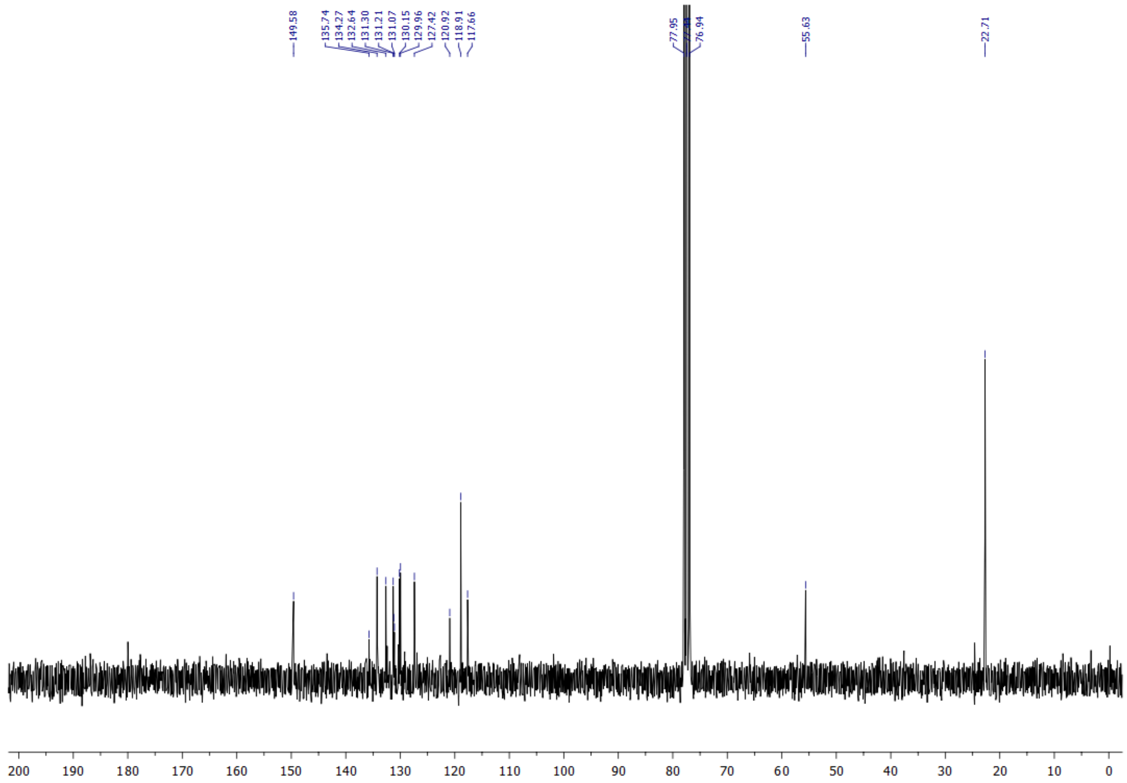
149.58
135.74
134.27
132.64
131.30
131.21
131.07
130.15
129.96
127.42
120.92
118.91
117.66
77.95
76.94
55.63
22.71
200
190
180
170
160
150
140
130
120
110
100
90
80
70
60
50
40
30
20
10
0

(*S*,*Z*)-*N*-((2-Amino-5-chlorophenyl)(2-chlorophenyl)methylene)-2-methylpropane-2-sulfinamide (***S*-1**)

^{1}H NMR (250 MHz, $CDCl_3$)

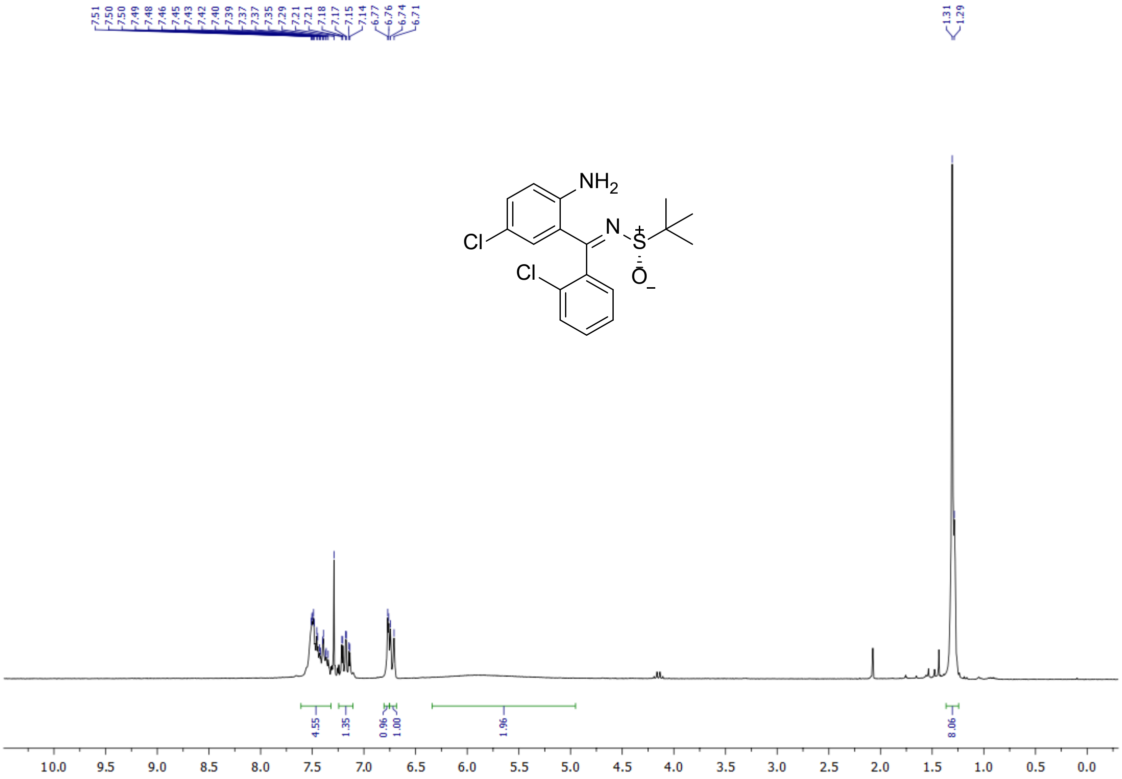

(*R*)-*N*-[(*S*)-(2-Amino-5-chlorophenyl)(2-chlorophenyl)methyl]-2-methylpropane-2-sulfinamide (***R*,*S*-2**)

^{1}H NMR (250 MHz, $CDCl_3$)

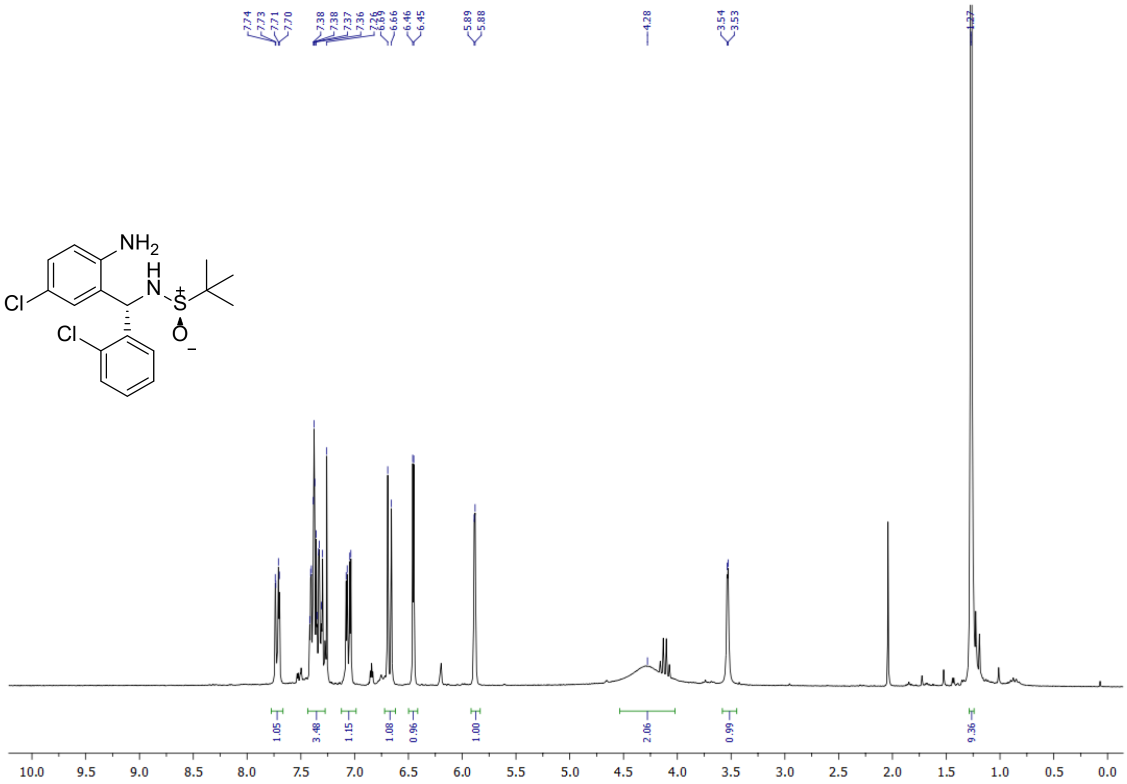

^{13}C NMR (63 MHz, $CDCl_3$)

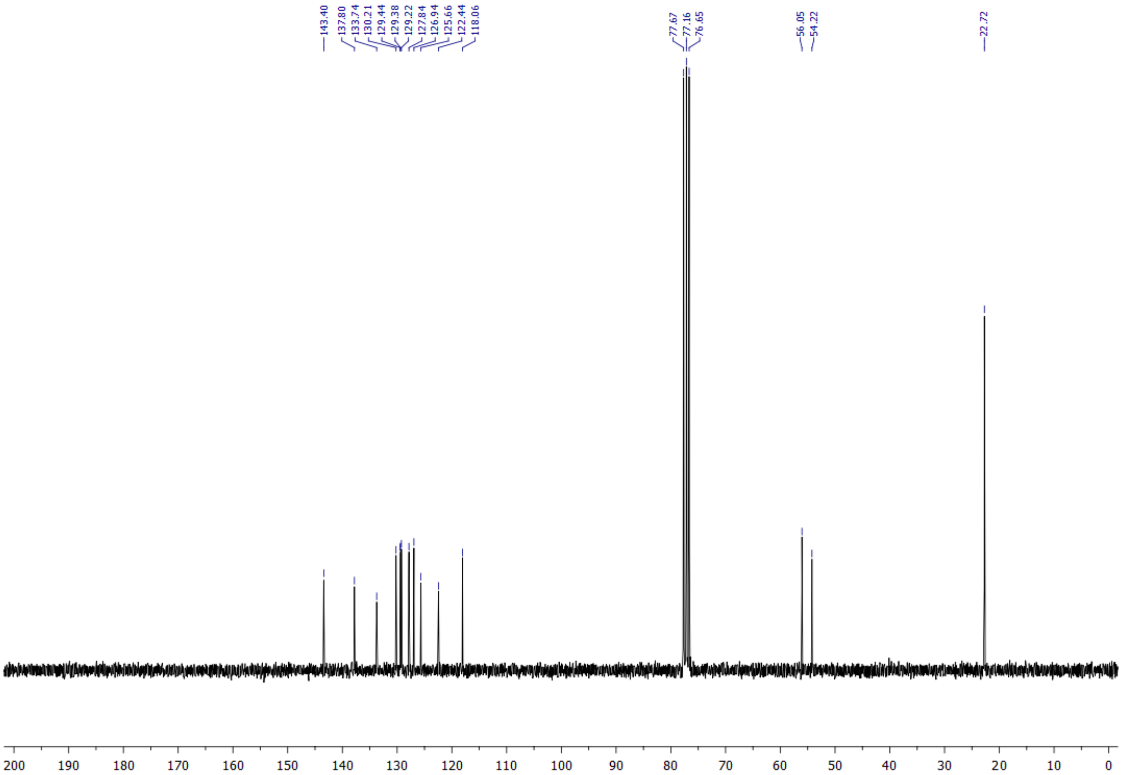

(*S*)-*N*-[(*R*)-(2-Amino-5-chlorophenyl)(2-chlorophenyl)methyl]-2-methylpropane-2-sulfinamide(***S*,*R*-2**)

^{1}H NMR (250 MHz, $CDCl_3$)

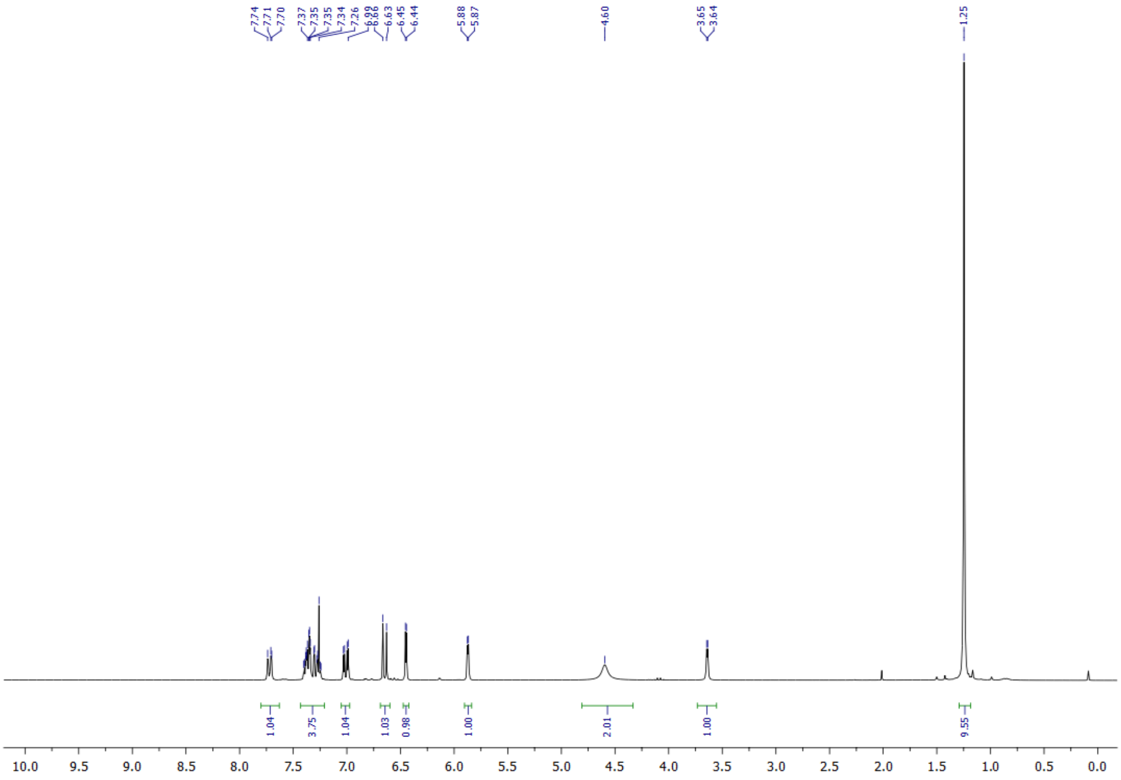

(*S*)-2-[Amino(2-chlorophenyl)methyl]-4-chloroaniline (***S*-3**)

¹H NMR (250 MHz, CDCl₃)

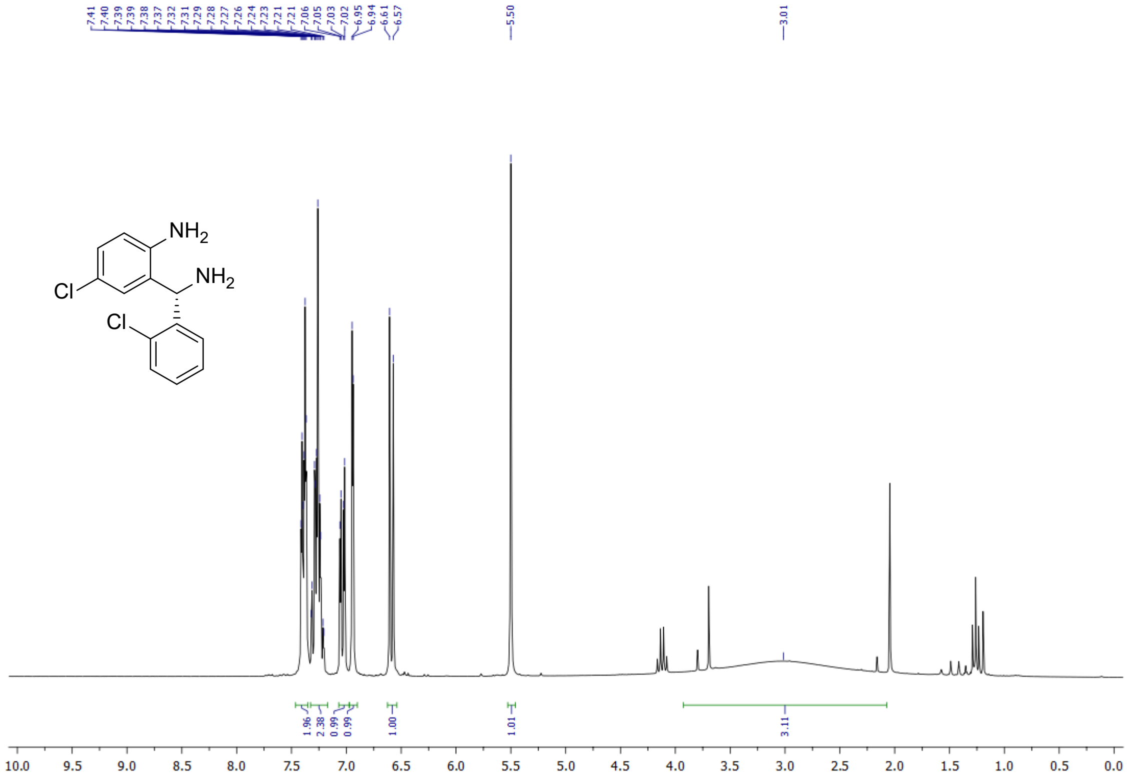

¹³C NMR (63 MHz, CDCl₃)

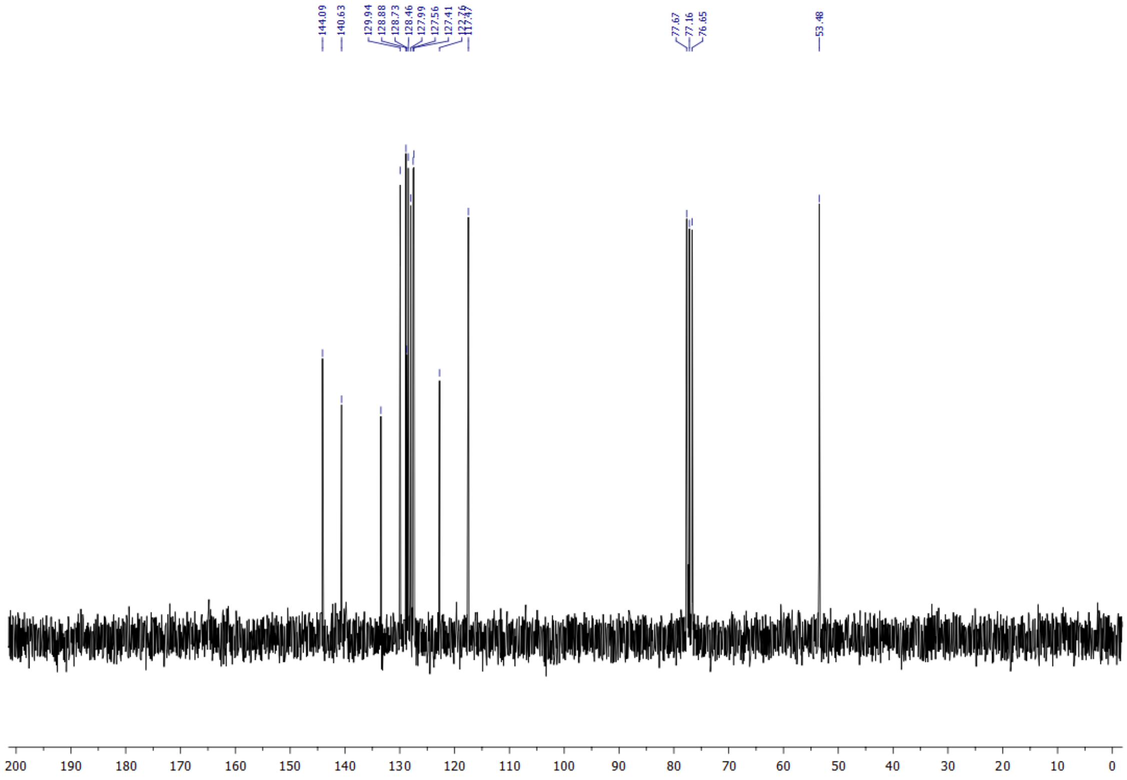

(*R*)-2-(Amino(2-chlorophenyl)methyl)-4-chloroaniline (***R*-3**)

^{1}H NMR (250 MHz, $CDCl_3$)

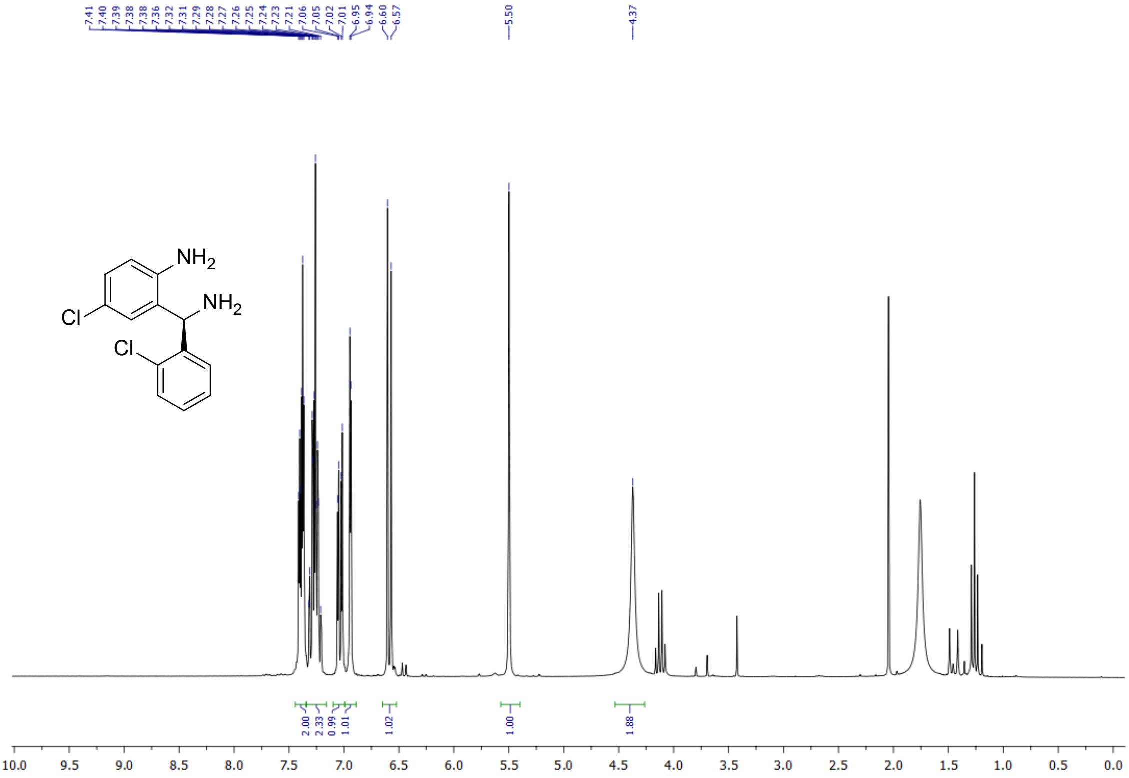

(*S*)-2-(((2-Amino-5-chlorophenyl)(2-chlorophenyl)methyl)amino)ethan-1-ol (***S*-4**)

^{1}H NMR (250 MHz, CDCl3)

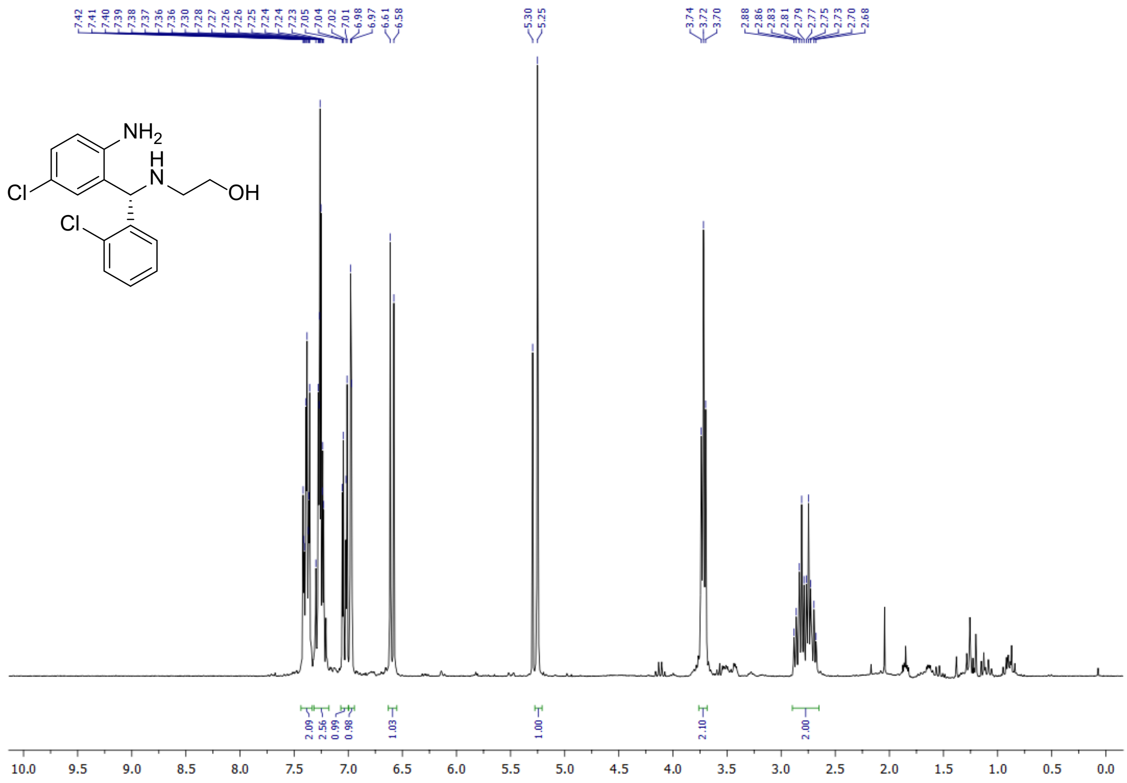

^{13}C NMR (63 MHz, CDCl3)

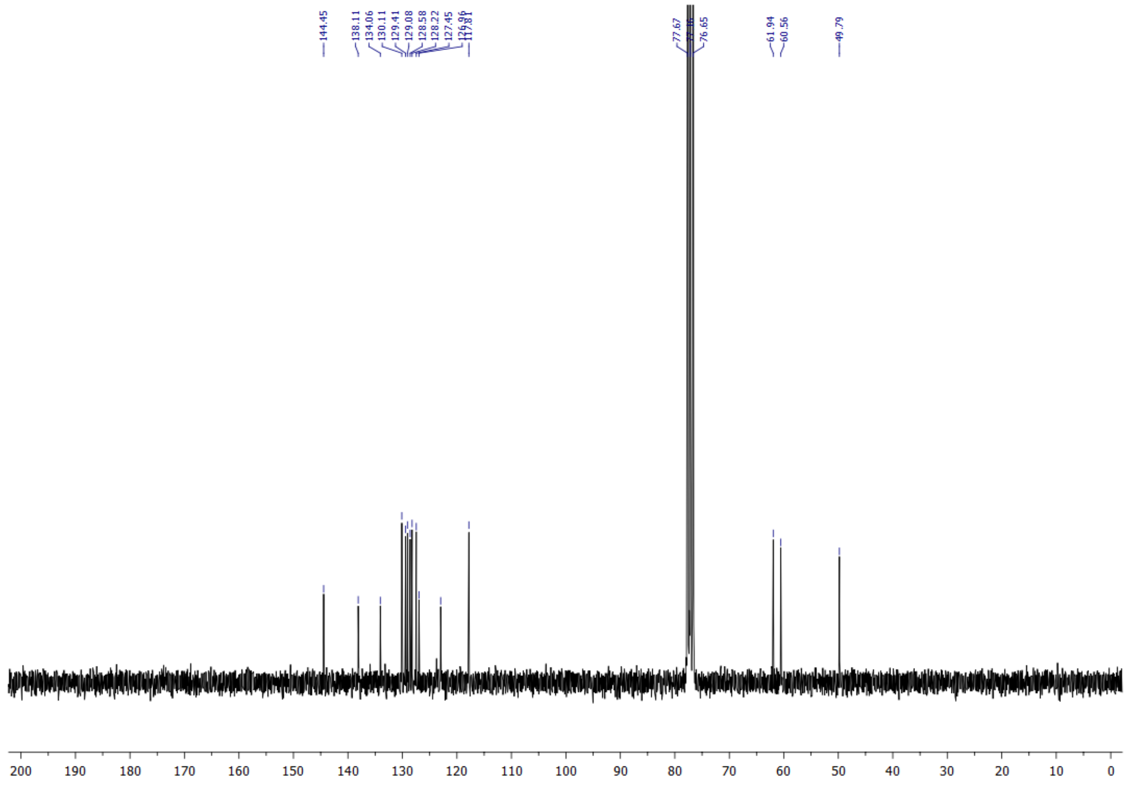

(*R*)-2-(((2-amino-5-chlorophenyl)(2-chlorophenyl)methyl)amino)ethan-1-ol (***R*-4**)

^{1}H NMR (250 MHz, CDCl3)

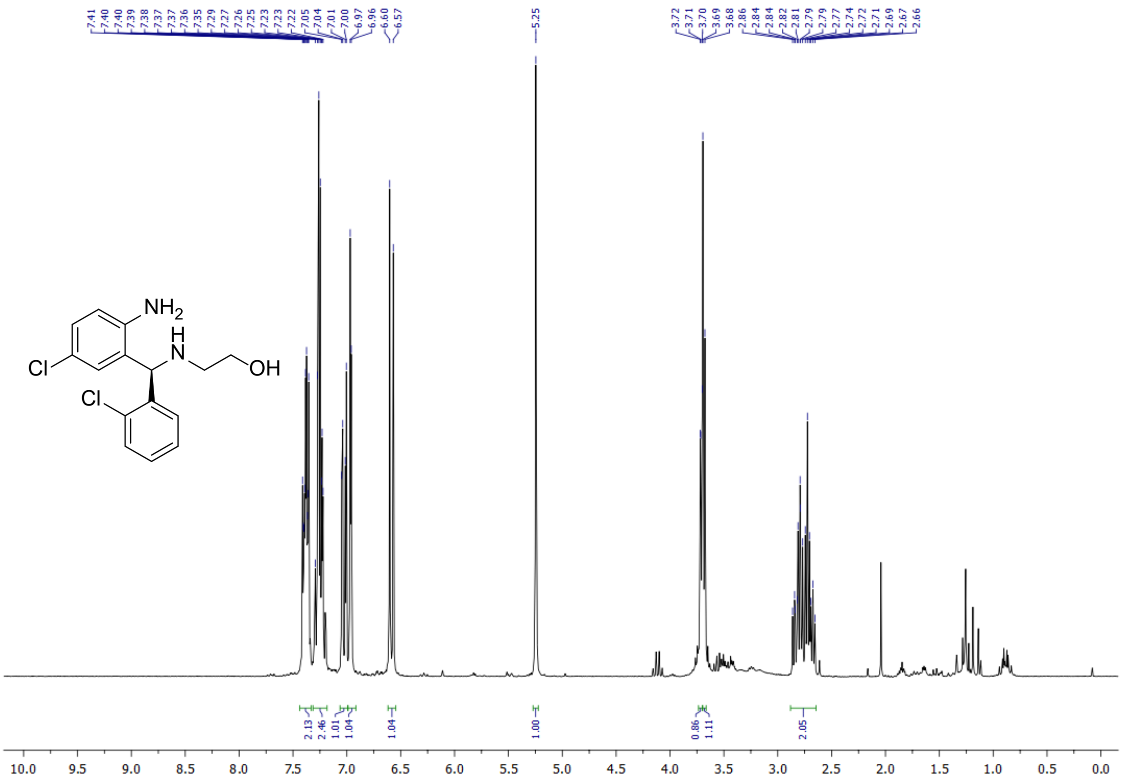

(*S*)-2-(7-Chloro-5-(2-chlorophenyl)-2-oxo-1,2,3,5-tetrahydro-4*H*-benzo[*e*][1,4]diazepin-4-yl)ethyl 2-bromoacetate (***S*-5**)

^{1}H NMR (250 MHz, $CDCl_3$)

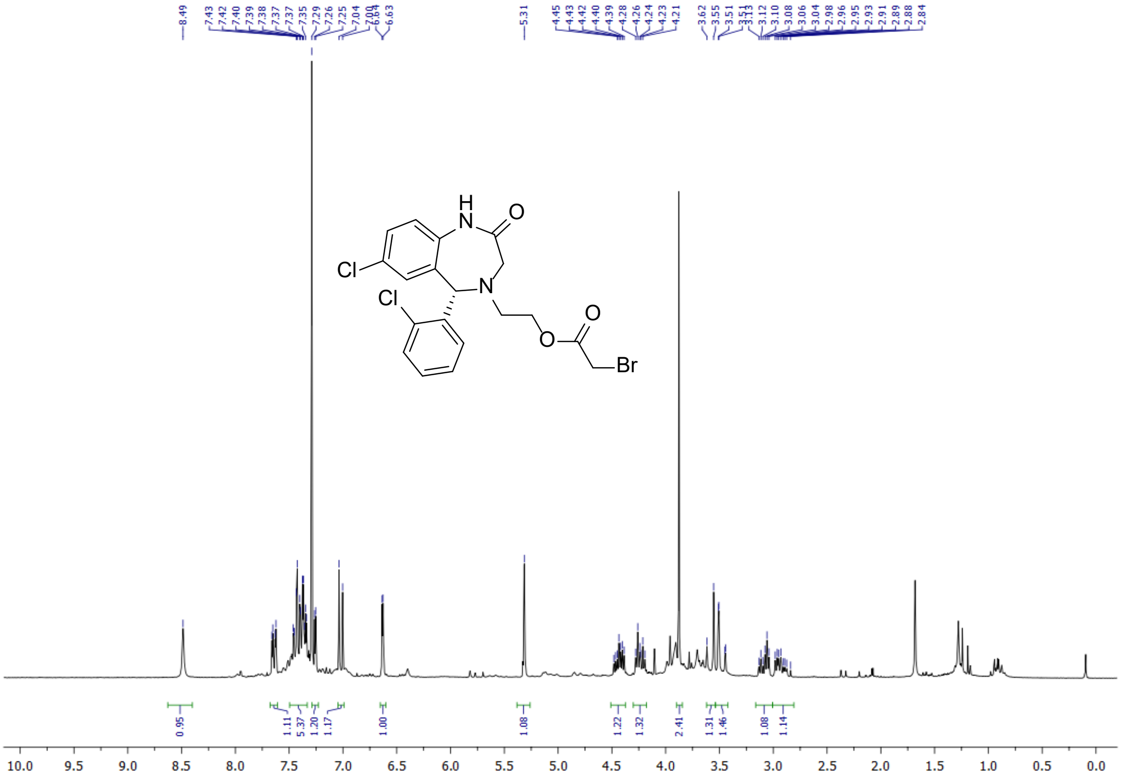

^{13}C NMR (63 MHz, $CDCl_3$)

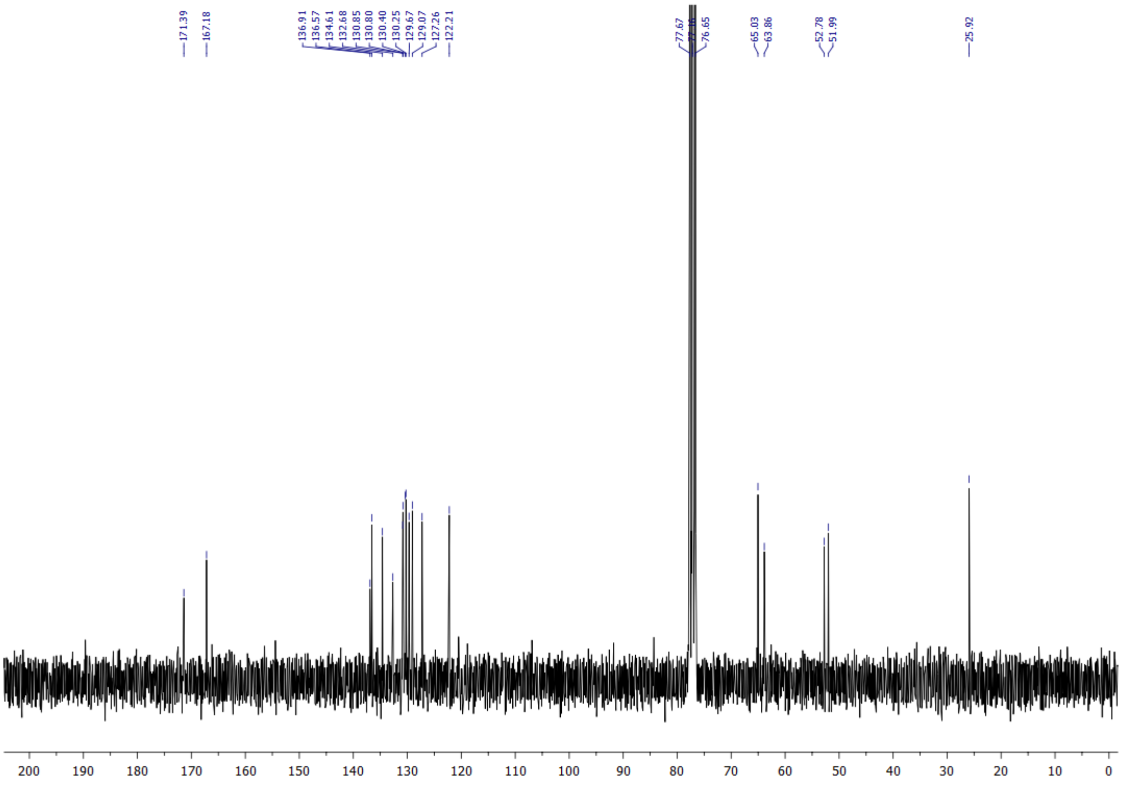

(*R*)-2-(7-Chloro-5-(2-chlorophenyl)-2-oxo-1,2,3,5-tetrahydro-4*H*-benzo[*e*][1,4]diazepin-4-yl)ethyl 2-bromoacetate (***R*-5**)

^{1}H NMR (250 MHz, CDCl$_3$)

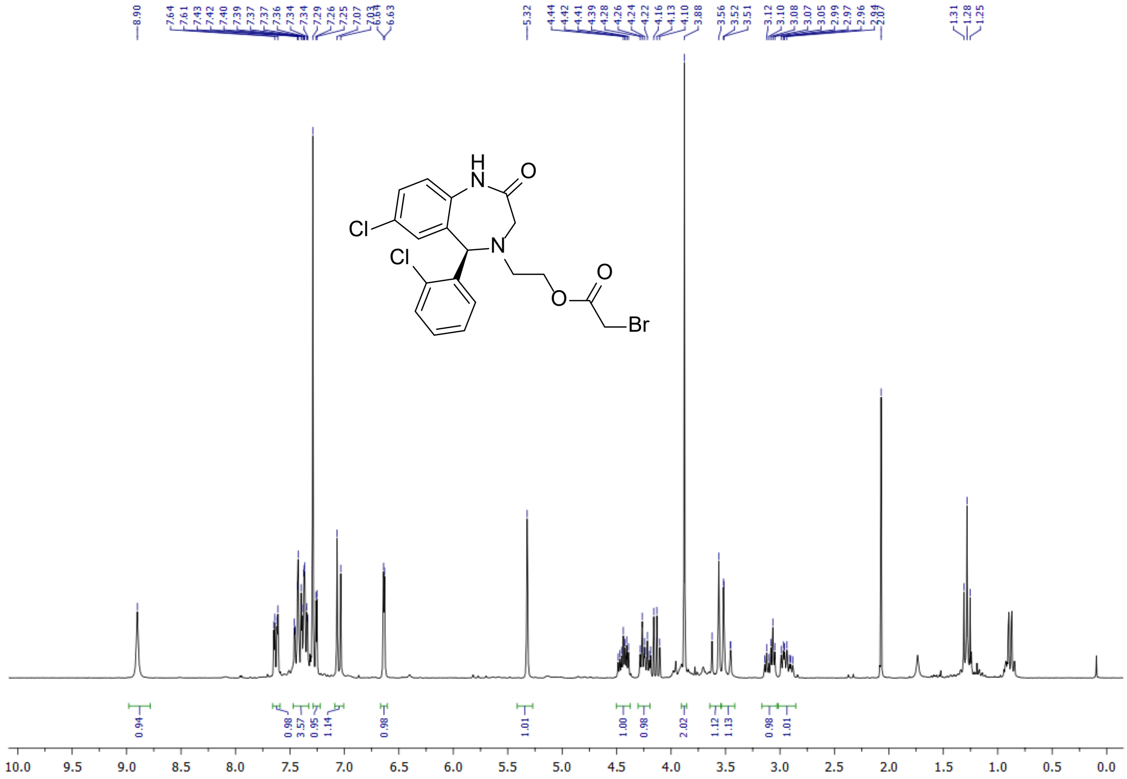

(5*S*)-7-Chloro-5-(2-chlorophenyl)-4-(2-hydroxyethyl)-4,5-dihydro-1*H*-benzo[*e*][1,4]diazepin-2(3*H*)-one (***S*-6**)

^{1}H NMR (250 MHz, CDCl$_3$)

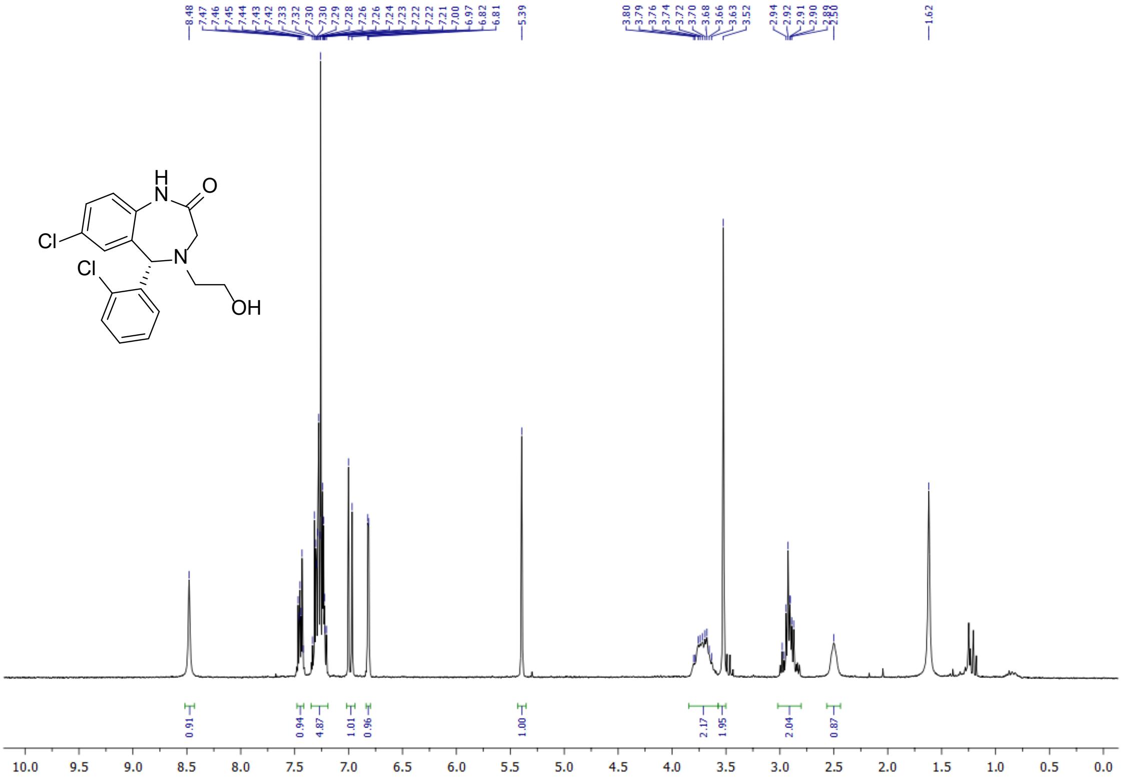

^{13}C NMR (63 MHz, CDCl$_3$)

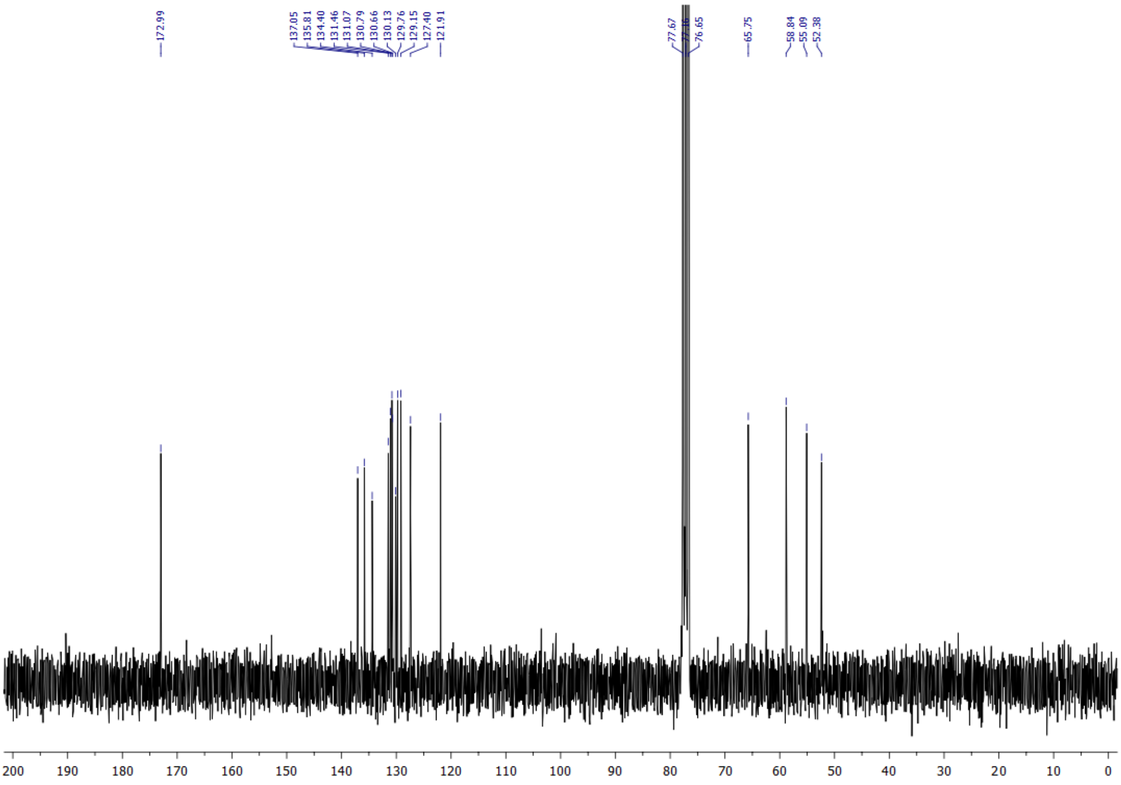

(5*R*)-7-Chloro-5-(2-chlorophenyl)-4-(2-hydroxyethyl)-4,5-dihydro-1*H*-benzo[*e*][1,4]diazepin-2(3*H*)-one (***R*-6**)

^{1}H NMR (250 MHz, $CDCl_3$)

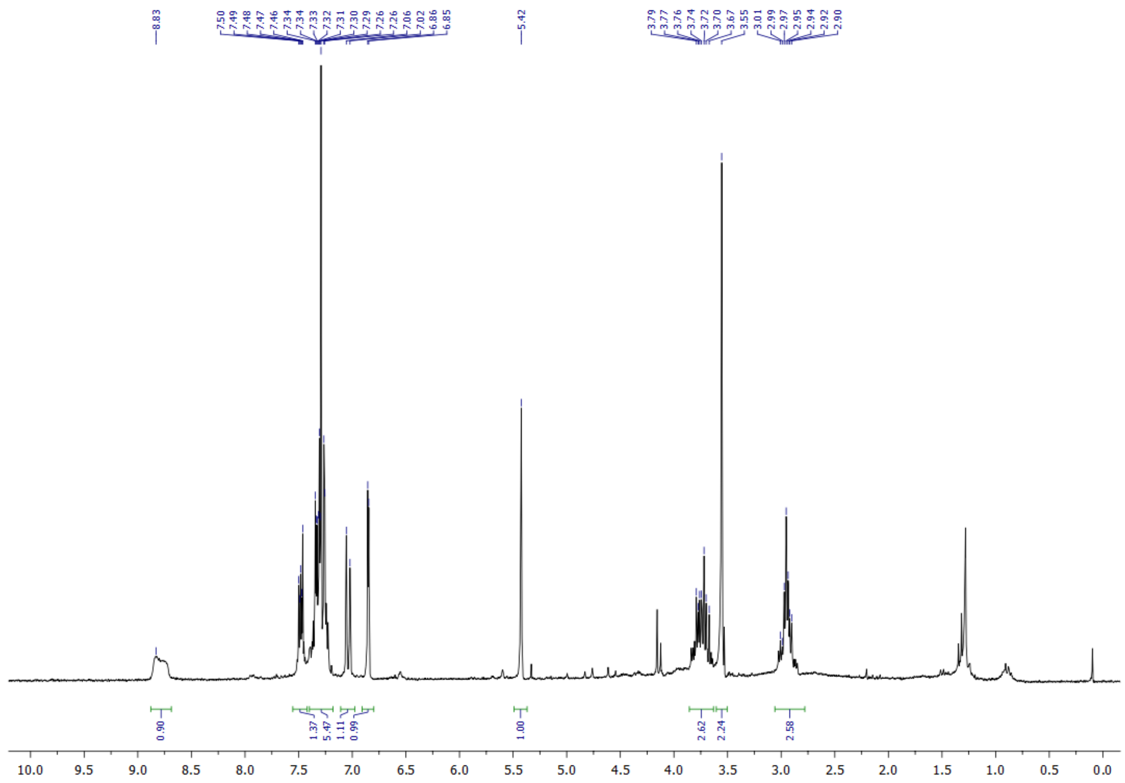

2-((*S*)-7-Chloro-5-(2-chlorophenyl)-2-oxo-1,2,3,5-tetrahydro-4*H*-benzo[*e*][1,4]diazepin-4-yl)ethyl 5-((*S*)-1,2-dithiolan-3-yl)pentanoate (**7a**)

^{1}H NMR (250 MHz, $CDCl_3$)

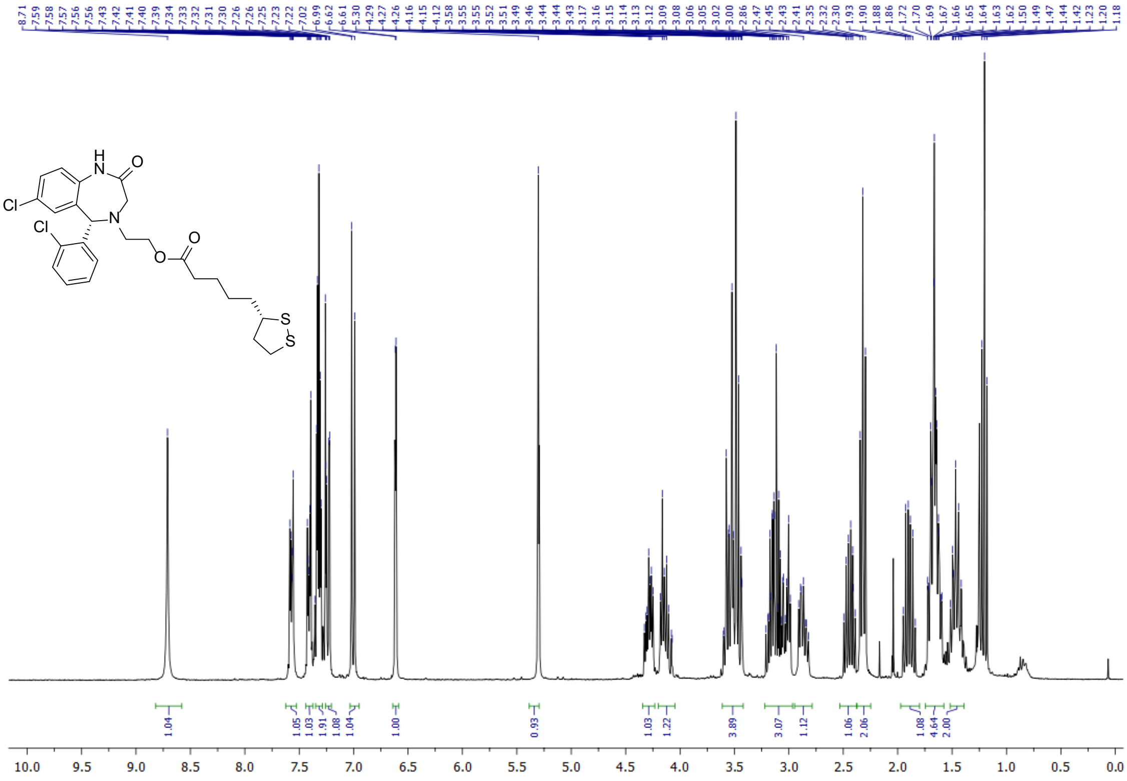

^{13}C NMR (63 MHz, $CDCl_3$)

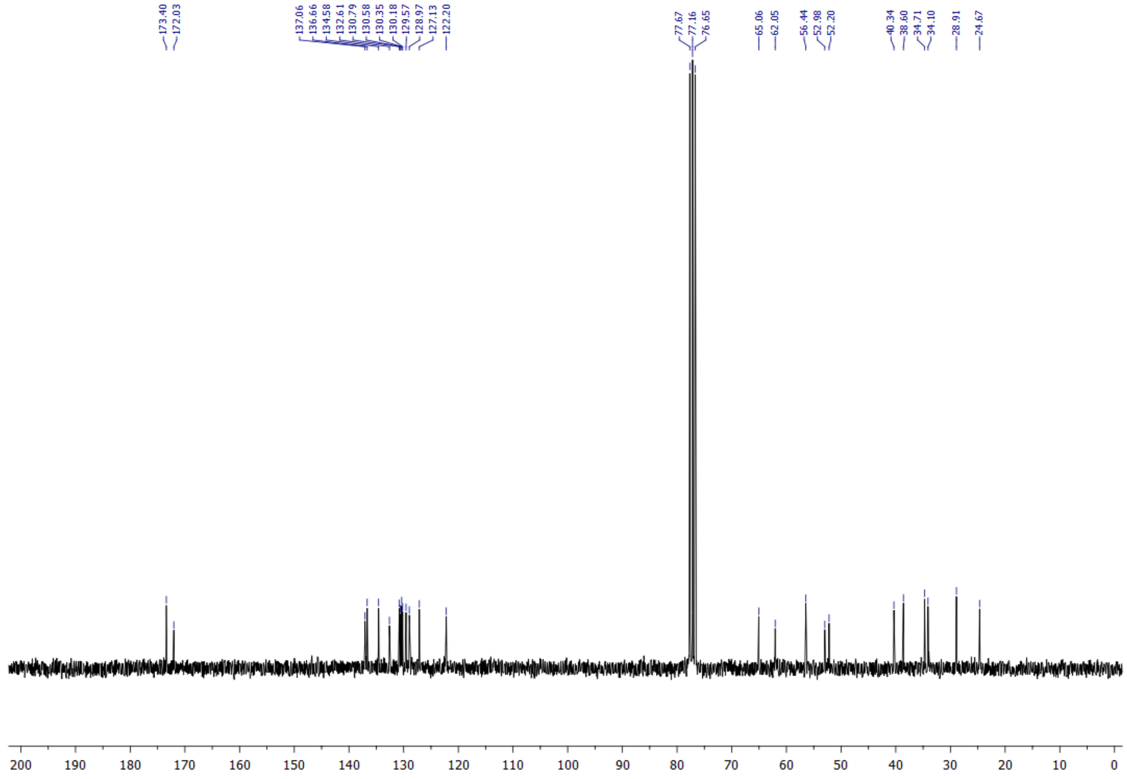

2-((*R*)-7-Chloro-5-(2-chlorophenyl)-2-oxo-1,2,3,5-tetrahydro-4*H*-benzo[*e*][1,4]diazepin-4-yl)ethyl 5-((*S*)-1,2-dithiolan-3-yl)pentanoate (**7b**)

^{1}H NMR (250 MHz, CDCl₃)

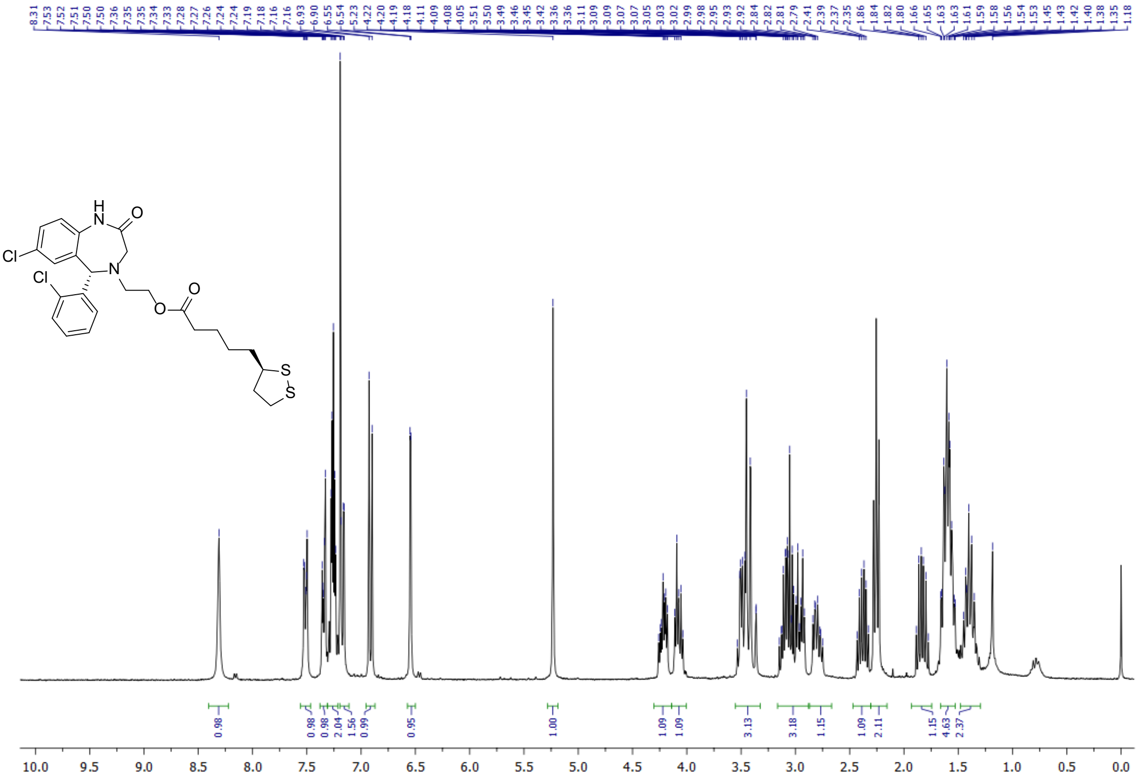

2-((*S*)-7-Chloro-5-(2-chlorophenyl)-2-oxo-1,2,3,5-tetrahydro-4*H*-benzo[*e*][1,4]diazepin-4-yl)ethyl 5-((*R*)-1,2-dithiolan-3-yl)pentanoate (**7c**)

^{1}H NMR (250 MHz, $CDCl_3$)

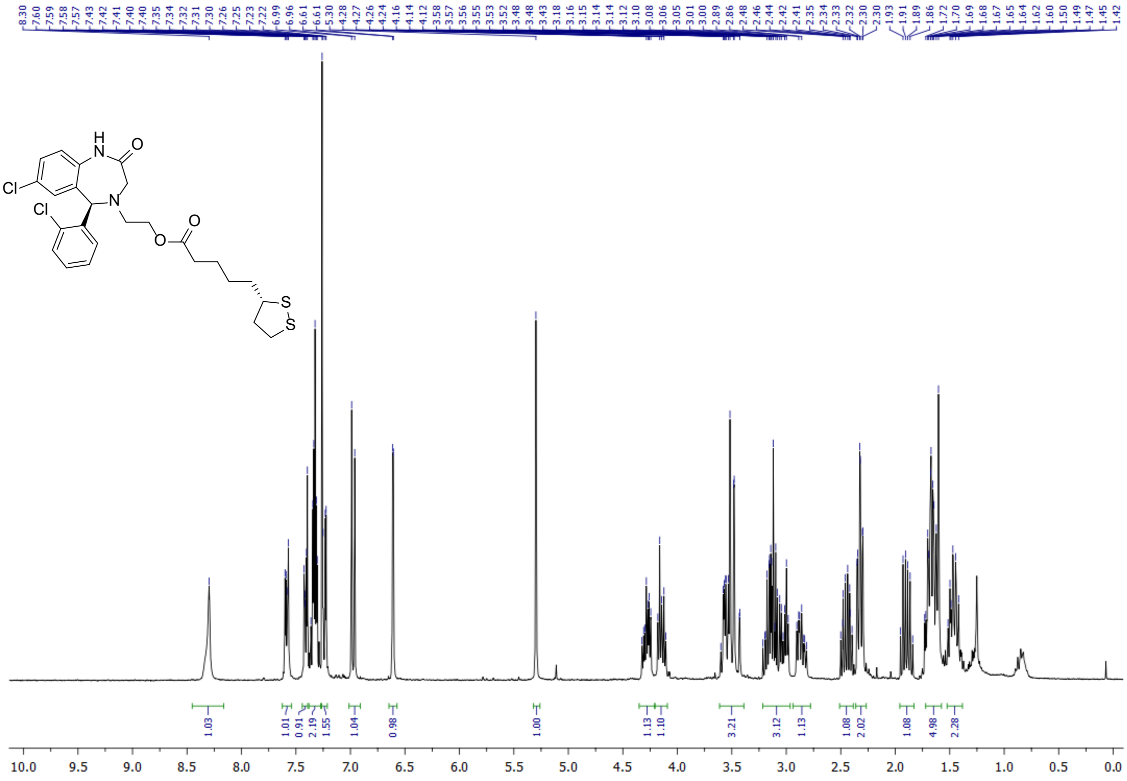

2-((*R*)-7-Chloro-5-(2-chlorophenyl)-2-oxo-1,2,3,5-tetrahydro-4*H*-benzo[*e*][1,4]diazepin-4-yl)ethyl 5-((*R*)-1,2-dithiolan-3-yl)pentanoate (**7d**)

^{1}H NMR (250 MHz, CDCl3)

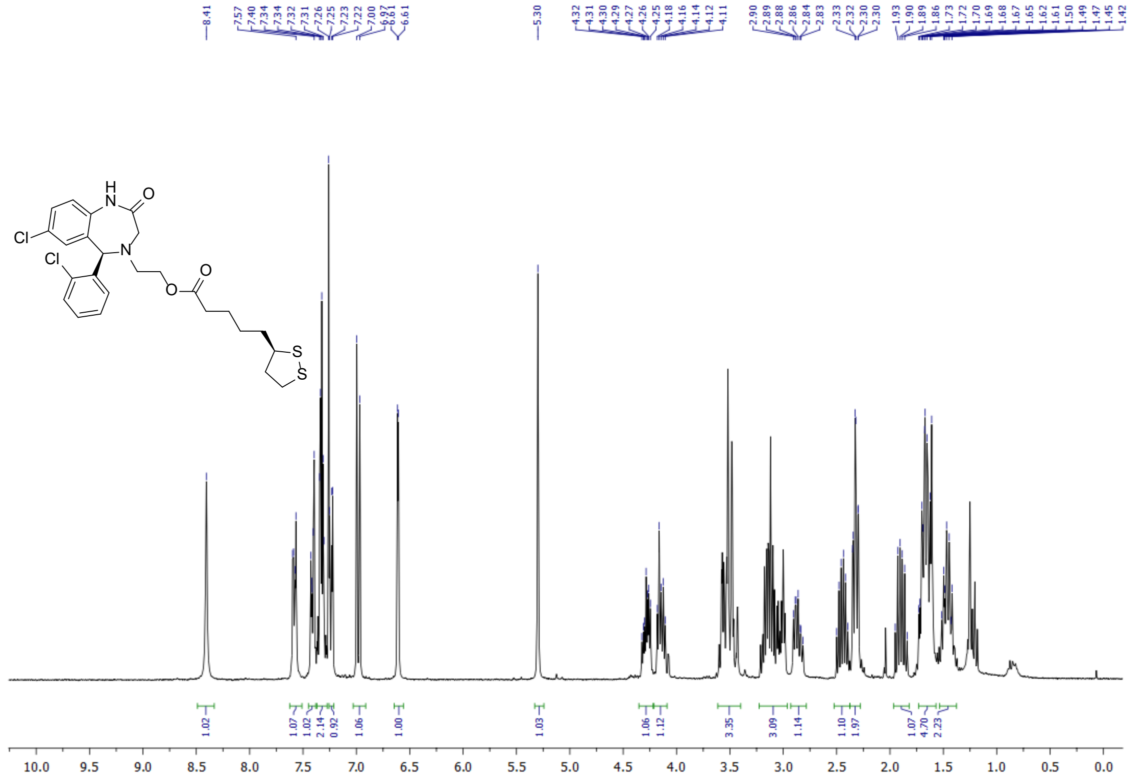

Copies of chromatograms

(rac)-2-(((2-amino-5-chlorophenyl)(2-chlorophenyl)methyl)amino)ethan-1-ol (***rac*-4**)

HPLC (ULTRON ES OVM Chiral Analytical Reverse Phase, 5µm, 4.6 mm x 150 L, buffer HNaPO4 20 mM pH 5.9/ CH_3CN 80:20, flow 1.0 mL/min.

Area % Report

Data File: D:\Enterprise\Projects\Default\Result\Diamina pH5.9_AcCN20%.rslt\Diamina pH5.9_AcCN20%.rslt.dat
Method: D:\Enterprise\Projects\Default\Method\Angel ph59-CH3CN20% 1ml.met
Acquired: 4/11/2018 2:01:30 PM (GMT +02:00)
Printed: 4/17/2018 4:23:56 PM (GMT +02:00)

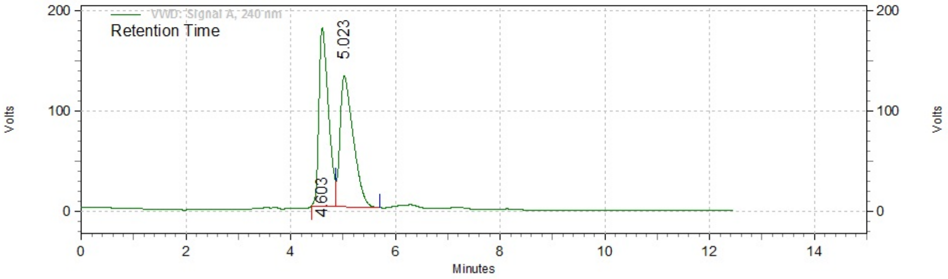

VWD: Signal A, 240 nm Results

Retention Time	Area	Area %	Height	Height %
4.603	37797369	49.52	2979248	57.70
5.023	38527866	50.48	2183790	42.30
Totals	76325235	100.00	5163038	100.00

(*S*)-2-(((2-Amino-5-chlorophenyl)(2-chlorophenyl)methyl)amino)ethan-1-ol (***S*-4**)

ee 93% The enantiomers were separated by HPLC (ULTRON ES OVM Chiral Analytical Reverse Phase, 5µm, 4.6 mm x 150 L, buffer NaH_2PO_4 20 mM pH 5.9/ CH_3CN 80:20, flow 1.0 mL/min.

Area % Report

Data File: D:\Enterprise\Projects\Default\Result\diasteroA_Diamina pH5.9_AcCN20%b.rslt\diasteroA_Diamina pH5.9_AcCN20%b.rslt.dat
Method: D:\Enterprise\Projects\Default\Result\diasteroA_Diamina pH5.9_AcCN20%b.rslt\Angel ph59-CH3CN20% 1ml.met
Acquired: 4/11/2018 2:37:07 PM (GMT +02:00)
Printed: 4/17/2018 4:26:54 PM (GMT +02:00)

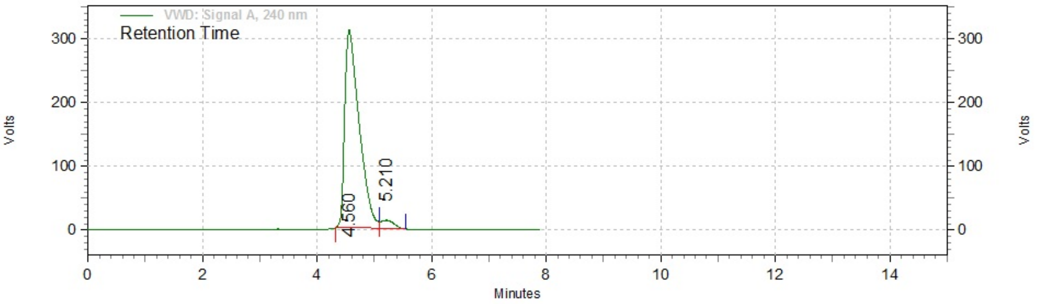

VWD: Signal A, 240 nm Results

Retention Time	Area	Area %	Height	Height %
4.560	91315109	96.35	5194146	95.97
5.210	3463364	3.65	217952	4.03
Totals	94778473	100.00	5412098	100.00

(*rac*)-7-Chloro-5-(2-chlorophenyl)-4-(2-hydroxyethyl)-4,5-dihydro-1*H*-benzo[*e*][1,4]diazepin-2(3*H*)-one (***rac*-6**)

Area % Report

Data File: D:\Enterprise\Projects\Default\Result\bzdrac3.rslt\bzdrac3.rslt.dat
Method: D:\Enterprise\Projects\Default\Result\bzdrac3.rslt\Angel ph59-iPrOH20% 1ml.met
Acquired: 3/7/2019 7:14:50 PM (GMT +02:00)
Printed: 7/31/2019 2:59:15 PM (GMT +02:00)

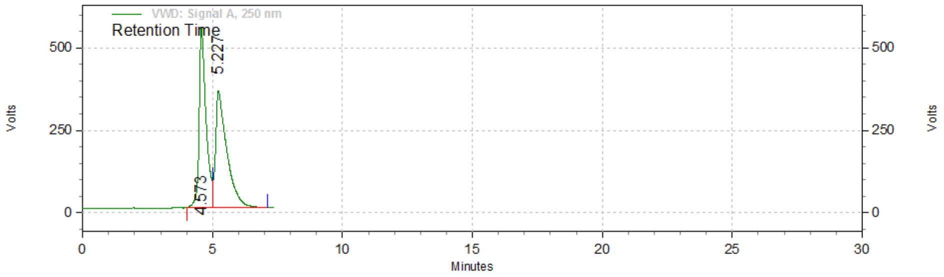

VWD: Signal A, 250 nm Results

Retention Time	Area	Area %	Height	Height %
4.573	167514860	48.44	9151422	60.66
5.227	178334005	51.56	5934470	39.34

The enantiomers were separated by HPLC (ULTRON ES OVM Chiral Analytical Reverse Phase, 5μm, 4.6 mm x 150 L, buffer NaH_2PO_4 20 mM pH 5.9/ *i*-PrOH 80:20, flow 1.0 mL/min.

(5*S*)-7-Chloro-5-(2-chlorophenyl)-4-(2-hydroxyethyl)-4,5-dihydro-1*H*-benzo[*e*][1,4]diazepin-2(3*H*)-one (***S*-6**)

ee 93% The enantiomers were separated by HPLC (ULTRON ES OVM Chiral Analytical Reverse Phase, 5μm, 4.6 mm x 150 L, buffer NaH_2PO_4 20 mM pH 5.9/ *i*-PrOH 80:20, flow 1.0 mL/min.

Area % Report

Data File: D:\Enterprise\Projects\Default\Result\ac102a.rslt\ac102a.rslt.dat
Method: D:\Enterprise\Projects\Default\Result\ac102a.rslt\Angel ph59-iPrOH20% 1ml.met
Acquired: 3/7/2019 8:23:51 PM (GMT +02:00)
Printed: 7/31/2019 3:13:07 PM (GMT +02:00)

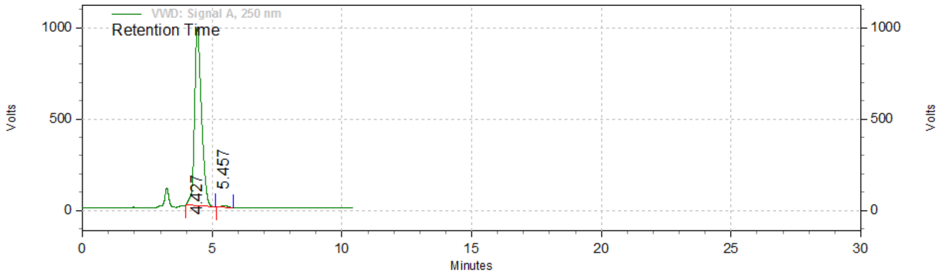

VWD: Signal A, 250 nm Results

Retention Time	Area	Area %	Height	Height %
4.427	306622680	99.04	16399498	98.92
5.457	2973713	0.96	178412	1.08
Totals	309596393	100.00	16577910	100.00

(5*S*)-7-Chloro-5-(2-chlorophenyl)-4-(2-hydroxyethyl)-4,5-dihydro-1*H*-benzo[*e*][1,4]diazepin-2(3*H*)-one (***R*-6**)
HPLC (ULTRON ES OVM Chiral Analytical Reverse Phase, 5µm, 4.6 mm x 150 L, buffer NaH_2PO_4 20 mM pH 5.9/ *i*-PrOH 80:20, flow 1.0 mL/min.

Area % Report

Data File: D:\Enterprise\Projects\Default\Result\ac101a.rslt\ac101a.rslt.dat
Method: D:\Enterprise\Projects\Default\Result\ac101a.rslt\Angel ph59-iPrOH20% 1ml.met
Acquired: 3/7/2019 8:08:44 PM (GMT +02:00)
Printed: 7/31/2019 3:07:30 PM (GMT +02:00)

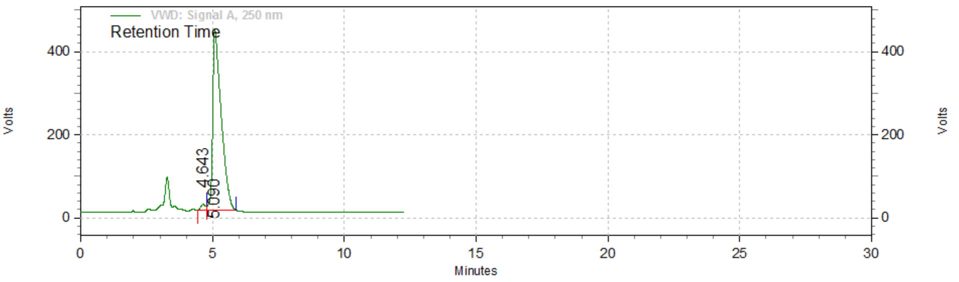

VWD: Signal A,
250 nm Results

Retention Time	Area	Area %	Height	Height %
4.643	3077942	1.84	249477	3.29
5.090	163830168	98.16	7326067	96.71
Totals	166908110	100.00	7575544	100.00

Copies of high-resolution mass spectra

2-((*S*)-7-Chloro-5-(2-chlorophenyl)-2-oxo-1,2,3,5-tetrahydro-4*H*-benzo[*e*][1,4]diazepin-4-yl)ethyl 5-((*S*)-1,2-dithiolan-3-yl)pentanoate (**7a**)

Comment 1 dctb
Comment 2 MeOH

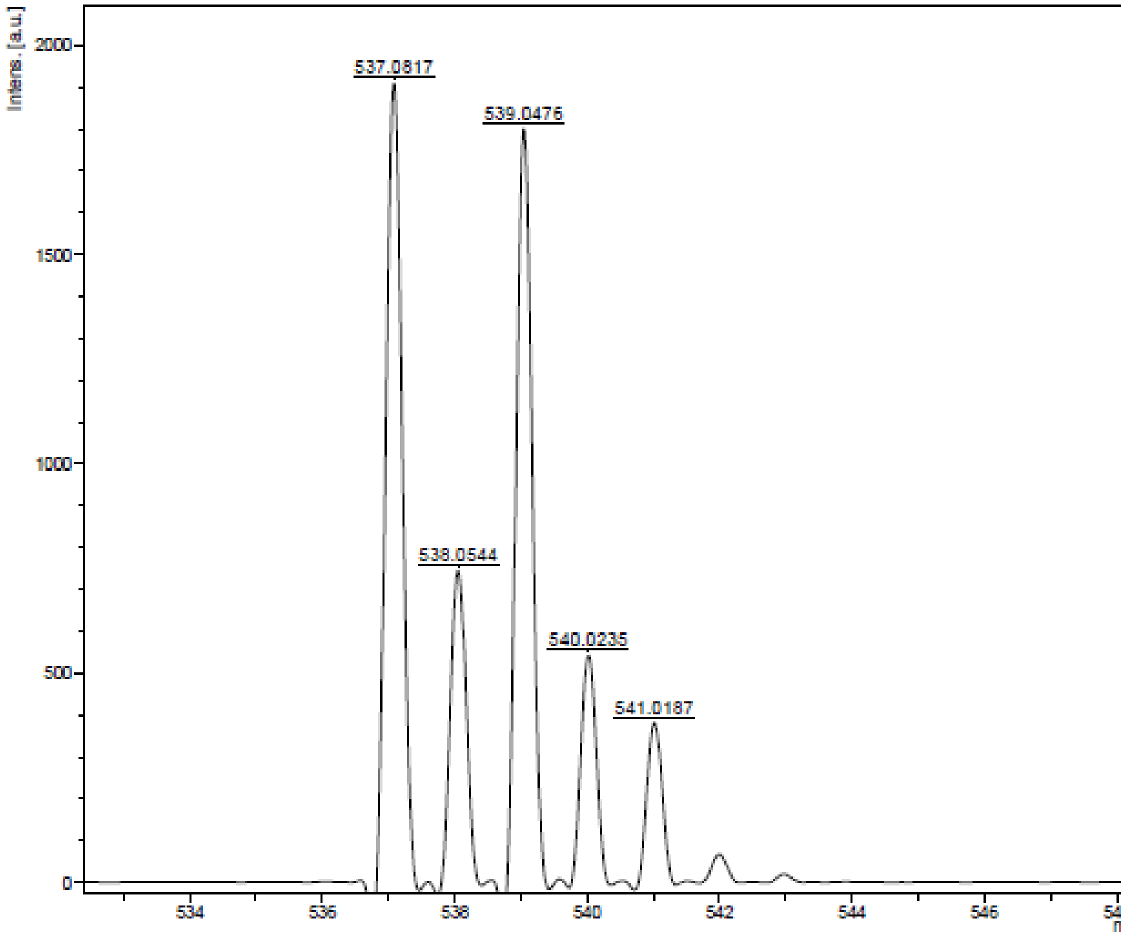

Acquisition Parameter

Date of acquisition	2021-12-22 12:54:17
Acquisition method name	D:\Methods\flexControlMethods\RP_Proteomics_HPC_20211222.par
Aquisition operation mode	Reflector
Voltage polarity	POS
Number of shots	200
Name of spectrum used for calibration	09-00-calpep\0_M4\2
Calibration reference list used	PeptideCalibStandard mono

2-((*R*)-7-Chloro-5-(2-chlorophenyl)-2-oxo-1,2,3,5-tetrahydro-4*H*-benzo[*e*][1,4]diazepin-4-yl)ethyl 5-((*S*)-1,2-dithiolan-3-yl)pentanoate (**7b**)

Comment 1 dctb

Comment 2 MeOH

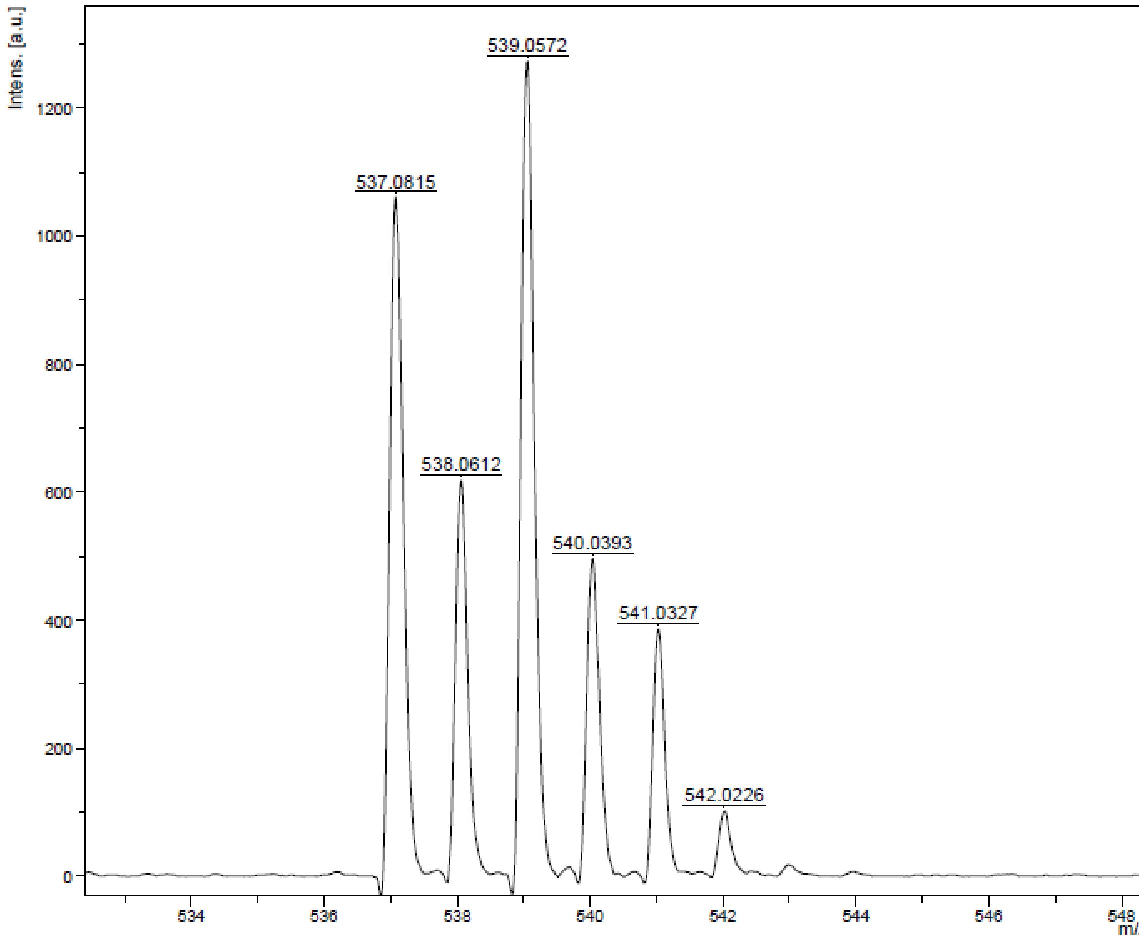

Acquisition Parameter

Date of acquisition	2021-12-22 12:54:39
Acquisition method name	D:\Methods\flexControlMethods\RP_Proteomics_HPC_20211222.par
Aquisition operation mode	Reflector
Voltage polarity	POS
Number of shots	200
Name of spectrum used for calibration	09-00-calpep\0_M4\2
Calibration reference list used	PeptideCalibStandard mono

2-((*S*)-7-Chloro-5-(2-chlorophenyl)-2-oxo-1,2,3,5-tetrahydro-4*H*-benzo[*e*][1,4]diazepin-4-yl)ethyl 5-((*R*)-1,2-dithiolan-3-yl)pentanoate (**7c**)

Comment 1	dctb
Comment 2	MeOH

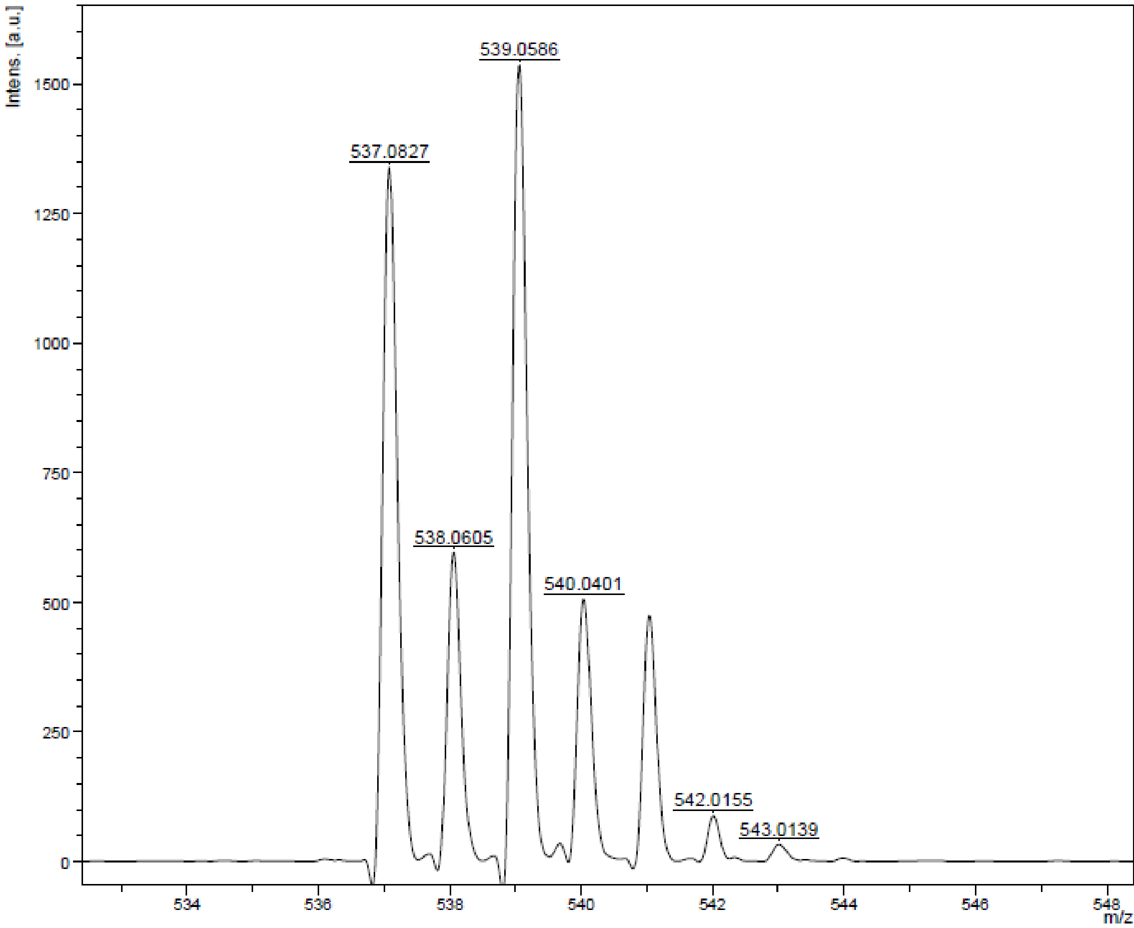

Acquisition Parameter

Date of acquisition	2021-12-22 12:54:53
Acquisition method name	D:\Methods\flexControlMethods\RP_Proteomics_HPC_20211222.par
Aquisition operation mode	Reflector
Voltage polarity	POS
Number of shots	200
Name of spectrum used for calibration	09-00-calpep\0_M4\2
Calibration reference list used	PeptideCalibStandard mono

2-((*R*)-7-Chloro-5-(2-chlorophenyl)-2-oxo-1,2,3,5-tetrahydro-4*H*-benzo[*e*][1,4]diazepin-4-yl)ethyl 5-((*R*)-1,2-dithiolan-3-yl)pentanoate (**7d**)

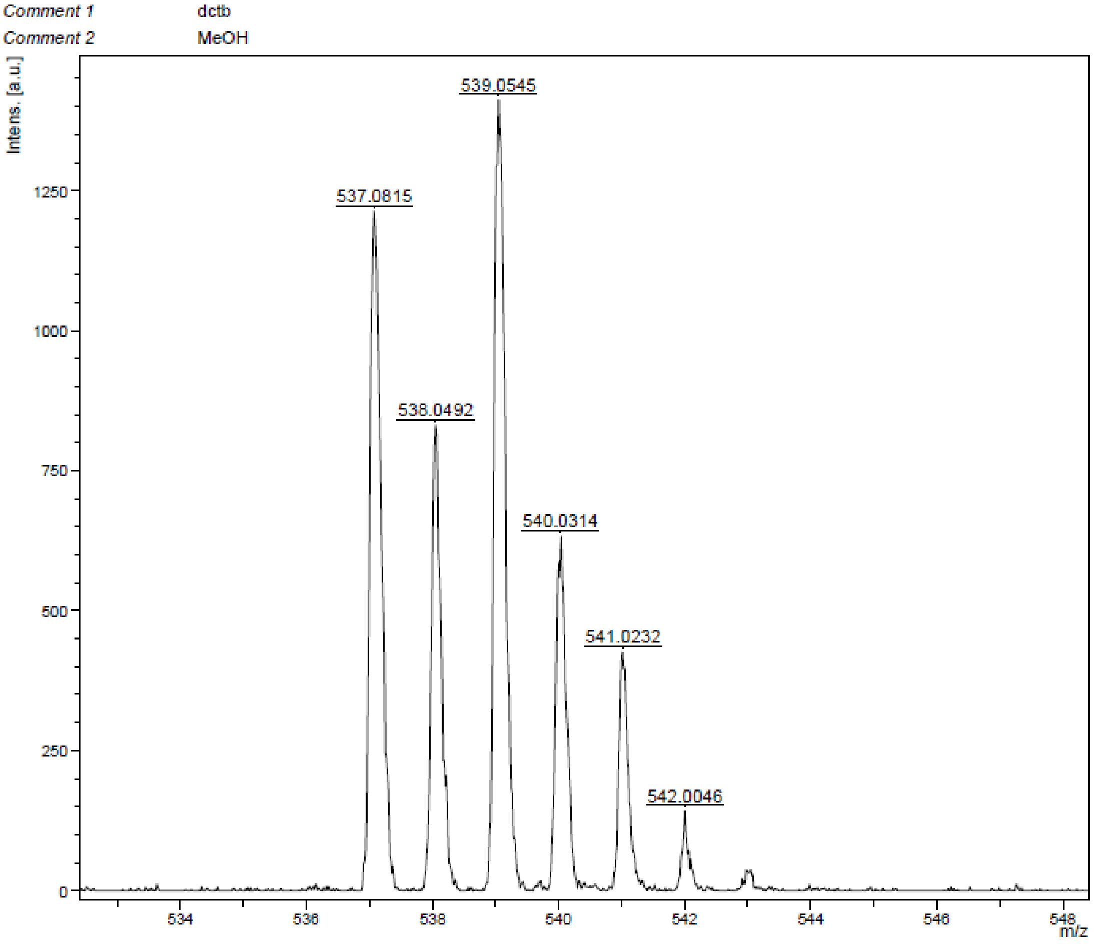

Acquisition Parameter

Date of acquisition	2021-12-22 12:55:09
Acquisition method name	D:\Methods\flexControlMethods\RP_Proteomics_HPC_20211222.par
Aquisition operation mode	Reflector
Voltage polarity	POS
Number of shots	200
Name of spectrum used for calibration	09-00-calpep\0_M4\2
Calibration reference list used	PeptideCalibStandard mono
